# Population Approaches to Promoting Relational Health From Early Life

**DOI:** 10.1007/s10567-026-00566-6

**Published:** 2026-03-16

**Authors:** Stephen R. Zubrick, Tracy Evans-Whipp, Craig A. Olsson, the Australian Early Relational Health Network

**Affiliations:** 1 Kids Research Institute Australia, The University of Western Australia, Perth, Australia; 2 Faculty of Health, SEED Lifespan Strategic Research Centre, School of Psychology, Deakin University, Victoria, Australia; 3 Department of Paediatrics, Centre for Adolescent Health, Murdoch Children’s Research Institute, The University of Melbourne, Royal Children’s Hospital Campus, Victoria, Australia

**Keywords:** Relational health, Early life, Population studies, Systematic reviews

## Abstract

In this paper we provide an integrative synthesis of eight systematic reviews that comprise our systematic review series entitled ‘*Population Perspectives on Nurturing Relational Health from Early Life*’. We reflect on what we know, what we don’t know, and what we need to know to better safeguard the interpersonal world of the child. Our review series brings forth evidence addressing a fundamental question: How do we create the basis for optimal development of relational skills that enable humans to establish trust, regulate their emotions, engage in exploratory behaviour, communicate effectively, apply introspection, and be more self-efficacious in meeting opportunities and challenges. The collaborating authors provide a unique opportunity to view the current status of this evidence across the bioecology of early human development. They find that our current view of relational health is limited. Much of the extant evidence is concentrated in the microsystem of early life, and it is overly focussed on the mother-infant dyad. Intergenerational findings from longitudinal cohorts point to additional influences on early relational health prior to conception extending back into the life histories of parents; however, these data remain exceptionally rare. There are also immense gaps in cross-cultural applications and extensions that measure, describe and inform the developmental model of relational health along with a glaring and urgent gap in the impacts of the digital social ecology on relational health. Pleasingly, evaluated interventions to promote and influence relational health exist and some are being implemented at scale. However, the outcomes of implementation are almost an after-thought in evaluation designs. We conclude by reflecting on the challenges faced by researchers, governments, policy makers, and agencies in moving relational interventions to scale, and particularly the challenges posed to their onward sustainability, in place, and more universally.

Quality relationships from early life are central to the development of foundational emotional, cognitive and behavioural skills that produce onward cooperation, compromise, collaboration and trust in the people around us, and in our wider communities, governments, and between nations. These skills and abilities are developed within the context of the family from the earliest moments in life and are progressively elaborated and used throughout the rest of life (Patton et al., 2018).

We commenced this series with the intent to describe what we know and what we don’t know about best designing public policy to nurture relational health from early life. We focused on promotion of early relational health at the population level rather than clinical treatment of relational ruptures (e.g. child neglect and abuse) within health services. We placed a high value on the need to assemble state-of-the-art, systematic scoping reviews, of the science on what matters, what works, and what can be upscaled to promote relational health from early life at a whole of population level.

Reviews in the series were supported by a new artificial intelligence-based platform, LitQuest (Fuller-Tyszkiewicz et al., 2025). This not only enabled timely completion of reviews, within tight funding resources, it also positions us to establish “living reviews”, which are distinguished by their ongoing and dynamic accrual of contemporary and relevant research findings that—in this case—advance the field of relational health. Our implementation found AI to be assistive in the saving of time, human resources, and costs associated with the screening phase. On average around 36% of retrieved literature required human screening to determine inclusion relevance.

Our search method has focussed particularly on recognising that early relational health has its determinants beyond the immediate circumstances of the mother-infant dyad and birth family. This is because the early and onward development of relational health in infants, young children and their primary carers has never been insulated from the effects that occur through education, community settings, social and friendship networks, and the socio-political mechanisms that prompt, facilitate or constrain human development. Nor have the foundations of early relational health ever been divorced from parents’ own life histories and lived experiences of close relationships with parents, extended family, friends and colleagues prior to becoming parents.

The studies included in this series reflect broadly on the corpus of available research on understanding and promoting the collective relational wellbeing of populations, starting from early life (National Academies of Sciences and Medicine, 2025). They provide a comprehensive picture of the global literature that is of direct relevance to government and community planners and leaders of universal services including health, education and employment who are working to strengthen our public institutions. In this final paper we present what we have learned and what remains to be known as well as what we are ready to do now to promote relational health from early life.

## What Have We Learned and What Remains to be Known

There are several overarching observations from the systematic scoping reviews in this series, each of which yields specific insights for future action and investment.

First, virtually all reviews in this series noted that the preponderance of population cohort studies and trials has focused on strengthening relationships from early life within the family microsystem, with a particular emphasis on the mother-infant/toddler dyad. Much less has been done on the father-child dyad and even less on the mother-father-child triad and other significant relationships within the broader family social network (e.g., grandparents). Furthermore, family microsystem interventions nearly all focussed on ‘fixing parents’ through education and skills training, with little or no consideration of targeting the broader environments that directly affect *how* parents raise their children and the circumstance in which they do this.

Research on the ecological settings around the young family and community is now needed to inform policy and practice. New research of particular value would include a focus on: (a) family relationships that extend beyond the mother, and include more research on fathers, grandparents, and other kin relationships and supports; (b) non-family microsystems that examine non-kin friendship networks and the broader community (e.g., neighbours, health professionals, child-care, mentors, coaches etc.); (c) the mesosystem of connections between microsystems (e.g., families’ connections with childcare, childcare’s connections with communities); (d) exosystemic policies that determine the quality of health, education and workplace settings that enable families to operate in ways that ensure parents have the necessary time with their children to support their healthy development (the structures and supports that “care for the adult carers of children”); and, (e) macrosystemic beliefs and values that shape all other ecologies of human development, with a particular focus on prejudice and discrimination related to gender, class and race.

Second, the evidence of continuity in both secure and insecure attachment status, between infancy and early childhood, is well established. Early attachment security in infancy is associated with later positive behaviours in early childhood. In contrast, early parental harshness is associated with later difficulties in peer and intimate relationship in adolescence and young adulthood (Painter et al., 2025). It is important to note that associations are small to moderate in size suggesting that pathways initiated in early life can be redirected; that there is opportunity across the formative years of the early life course to transform insecure early relationships into secure ones (Allen et al., 2026). However, little is known about the longer-term effects beyond childhood, with the longest studies only extending as far as young adulthood, and no evidence into middle and later adult life.

New research should be initiated on interventions to strengthen relational health beyond the first year of life. In the first instance, such research could maximise the value of existing cohort data by modelling the developmental pathways that lead to relational difficulties and disfunction over childhood and onward into adolescence (see Macdonald et al. (2025)). This would support the call for reliable and continued research investment, from both government and non-government funding agencies, to ensure on-going follow-up of our longest running early life cohorts in to middle and later adulthood.

Third, existing mature cohort studies with intergenerational data and long-term follow up are showing that, beyond the influences of contemporaneous social ecologies, there exists an additional influence on early relational health prior to conception that extends back into the life histories of parents. Findings from intergenerational studies are now empirically measuring the extent to which experiences prior to becoming a parent are shaping the relational quality (for better or worse) of the family microsystem on having children. The types of intergenerational factors examined to date include early experiences in close relationships within family, friendship and education microsystems, histories of mental health strengths and difficulties (including substance use), persistent social economic disadvantage, and major life events including diagnosis of significant physical illness. Each has the potential to shape later capacities to build secure relationships with offspring once becoming parents (Macdonald et al., 2025).

This work invites new research to guide intergenerational policy design and interventions that build and strengthen relational health through the preconception period to early childhood and onward to young adulthood. As Macdonald et al. (2025) in this series note, the intergenerational literature in this regard is scant and nascent. Of value would be international studies that widen the view of the social ecology, are more inclusive of fatherhood, that better harmonize and invite integrating data across existing cohort studies to improve power and prevalence characteristics, and finally better target policy and practice applications.

Fourth, over 50 years ago Uri Bronfenbrenner recognised the need for cross-cultural studies to offer validation of universal developmental principles as well as counterfactual evidence to balance “mono-cultural” evidence (Bronfenbrenner, 1970). Yet available research about early relational health has been overly concentrated in Western, high-income countries leaving a vast developmental “blind spot” in knowledge and in scientific cross-cultural developmental science.

In their unique scoping review (this series), McNeair et al. show how Indigenous relational knowledge systems align with bioecological models and offer both validation for, and counter-factual evidence of, universal developmental principles (McNeair et al., 2026). The authors highlight key differences in the cultural form and expression of kinship and belonging, cultural identity, and autonomy and self-reliance that arise from collectivist relational systems within Australian Aboriginal culture. These are contrasted with the dominant Western developmental paradigm that has valorised relationships with human entities, principally dyadic relationships and nuclear family structures along with an emphasis on direct observational methods that restrict the relational lens.

The need to preserve Indigenous cultural knowledge around raising relationally heathy children is urgent (Dudgeon et al., 2023). McNeair et al. demonstrate that this is more than possible and how. The authors call for culturally valid, strengths-based tools that encompass the kinship structures, collective parenting norms and inter-generational contexts that characterise Australian Aboriginal culture (McNeair et al., 2026).

Fifth, our review series documents that numerous universal programs have been developed and evaluated to address many of the ecological determinants of early relational health identified in cohort studies. Many interventions demonstrate short-term positive effects on mother-infant, parent-parent, and to a lesser extent on father-infant relationships (Fuller-Tyszkiewicz et al., 2026). However, the same limitations noted above with aetiological research also apply to intervention development, with most focusing within the microsystem of the family (retaining a focus on mother–child relationships). Few extend to other microsystems, to building a strong mesosystem of connections between microsystems, to ensuring exosystemic institutions and structures that care for parents are further improved, and that seek to change the hearts and minds of people in ways that privilege, support, and give status to the roles of all those involved in raising the next generation.

Intervention development now needs to extend to all ecologies of human development, including those that focus on breaking intergenerational cycles of risk. This means that ‘silver bullet’ approaches to implementing interventions should be supplanted by multisystem investments that orchestrate interconnected interventions across ecological systems, starting with enriching mesosystemic connections between microsystems of family, childcare and community.

Sixth, there is a glaring gap in knowledge around the digital social ecology. This is emerging as an important influence set to shape human development, and one that is about to be supercharged through application of Gen-AI. A place of positive relationships where many communities and connections now form, and where networks and relationships are maintained, it is also a place where the influence of propaganda and trolling are resetting norms and values within a society over short periods of time.

Digital interaction has fast become central to the administration of most major social institutions (health, education, employment), changing the nature of our relationships with our service providers. Digital platforms also provide new points of information and connection within and across families, early education and community settings, and creates “techno-ference” which has the potential to disrupt parent–child interactions by distracting parents (Navarro & Tudge, 2023).

In comparison to corporate research and targeted studies, many long-term research initiatives around children and families have not adapted to measure or assess the impact of these new digital realities. Research investments are now needed to augment existing longitudinal studies, and to design new cohort studies, with contemporary measures of the digital social ecology to capture this key sociohistorical development. Furthermore, research on how the digital social ecology is shaping all other traditional ecologies of human development needs to be made quickly available to government to best inform approaches to maximise the benefits and mitigate any toxic effects of this new global ecology.

Finally, across many of the reviews in this series, calls were made for improvements in study methodologies for relational health. Two improvements are prominent. First, while intervention effects in RCTs affecting relational health are demonstrated, the report of implementation outcomes is almost an after-thought, with only 13% of criterion studies reporting these as part of their main effects (Pearce et al., 2026). The evidence base for what happens in the act of implementation remains scant with a need for explicit theories, models, frameworks, and standardised measures for evaluating implementation outcomes. Second, there is a consistent call to widen the prevailing view of relational health by utilizing existing longitudinal data and developing new data designs more inclusive of the wider social ecology. The predominate view in existing studies is heavily focused at the “micro” level. Studies that permit greater estimations of the effects of the meso-, exo-, and macro-systems are needed. So too, are mixed method approaches to understand the contribution of historical events (e.g. time and the chronosystem).

## The Onward Context for Sustainable Implementation

Our review series makes evident that researchers, governments, and service providers are already in action “doing” universal interventions affecting the development of relational health. There are certainly evaluated interventions ready to implement at scale (Allen et al., 2026; Fuller-Tyszkiewicz et al., 2026).

However, what also needs to be made ready in pursuing these opportunities are the frameworks for sustainability of interventions. Human development does not progress by “turning it on and off.” This imparts a level of chaos in the lives of children, their families and communities that is known to be counterproductive, if not outright damaging, to establishing well-regulated developmental growth, and within it, relational health (Evans & Wachs, 2010). In establishing sustainable institutional arrangements researchers particularly will need to assess both the relevance of their place and their role in this process. The employment life cycle and advancing research programs of most researchers, and to some extent, their roles and interests, lack the range of skills and time horizons for undertaking the establishment of the long-term institutional governance required to secure ongoing intervention implementation.

To do this will require a greater focus on *sustainable* forms of translation through institutionalised universal (government funded) platforms. The circumstances shaping these current opportunities set both constraints and define new expectations for how researchers, governments and services collaborate in moving interventions to scale.

Presently, in martialling limited funding and services for children and families, the modus operandi of jurisdictional and federal governments is one in which government policies and services seek to target those most in need. These services cater predominately for children who face developmental challenges, and those families in otherwise overwhelmed circumstances. While this “targeted” intervention strategy is regarded as morally responsible and economically defensible, the coverage of the target population is acknowledged to be fragmentary, inequitable and incomplete.

Consequently, government policies and services do not address the substantial *marginal gains* to be made in Australian human development for those children in the families of the working poor and with low human capital that lag consistently behind in their development and go on to have diminished life choices. It is these very circumstances that are more amenable to the effects of “universal” intervention strategies (Zubrick et al., 2022).

Furthermore, focusing efforts on high-risk settings ignores structural constraints that entrench, rather than resolve place-based inequities in populations. These constraints entail elements such as legislations, workplace regulations, social benefits, built environments, and the distribution and equity of services that affect the choices people can make. Whole-of-population approaches to promoting early relational health, that are delivered universally across populations proportionate to need, have the overall effect of ‘moving the mean’ of populations towards better relational health, which in turn, reduces the proportion of the population at risk in the ends of the distribution, where relational ruptures have a very significant toll on society (Rose, 2001).

The limitations of existing “targeted” approaches underlie, to a greater extent, the rise in community-led, place-based coalitions now seeking to address more universal child and adolescent development challenges in their communities by co-designing, scaling up, and implementing selected population based early relational health promotion interventions. These initiatives offer critically important opportunities to widen developmental intervention options for young populations. The enthusiasm of governments, non-government providers and researchers is evident (Geatches et al., 2023). However, the evidence base on place-based initiatives remains inconsistent, insufficient or isolated to the specific contexts with inadequate attention on addressing key features affecting program effectiveness and sustainability (Glover et al., 2021; Olfert et al., 2014).

There are several features in the context for wider implementation of universal interventions affecting the development of relational health that need to be addressed.

First, while there is an established scientific consensus about the effective “social ingredients” in early child development interventions (Duncan et al., 2022; Karoly, 2002; Karoly et al., 1998; Ramey & Ramey, 1998), the balance of findings would suggest that our expectations about implementing co-designed evidence-based interventions in place, relying on models using participatory action research, are overly optimistic. Australian research to date suggests that community development is a central component, but not sufficient itself to establish and sustain child development opportunities nor to systematically change child development trajectories (Crow et al., 2004; Edwards et al., 2014; Kellock, 2007).

Second, moving evidence-based methods to population-scale substantially weakens their effects (Duncan et al., 2022). Some of this effect-weakening is likely to occur owing to improvements in human services and education rather than from exposure to interventions per se; for example, Australia has extended the period of mandatory education from 10 years to 12 and widened access to early childhood education (Australian Bureau of Statistics, 2023a, b). But there are also persistent theory and execution failures in implementation at scale (European Monitoring Centre for Drugs & Drug Addiction, 2017). Anecdotal experience would also suggest that place-based knowledge is over-valorised relative to evidence-based knowledge in decisions about what works and how to select and implement it.

Third, as Allen et al. note (Allen et al., 2026), “the process of scaling up should incorporate sophisticated governance arrangements. There are interventions ready to take to scale. What needs to be made ready in pursuing these opportunities are the frameworks for sustainability.”

However, current models of community-led, co-designed initiatives are heavily reliant on local champions, loose coalitions of service providers, and State and Commonwealth leveraged funding. Evaluations of community-based participatory models reveal these models are inherently unstable and struggle to maintain long-term sustainability (Rowland et al., 2018). Moving available interventions to scale will require new institutional arrangements to: ensure that the factors which build and sustain relational health outcomes are embedded when scaling beyond community-services; recruit long-term Board members; fund project staff to provide training and induction; and employ a project coordinator to ensure fidelity and maintain motivation and enthusiasm (Crow et al., 2004; Kellock, 2007).

Institutional leadership, authoritative governance, ownership and accountability for outcomes are critical to sustaining the success of rolling out interventions where improvement in human development is being sought. Moreover, community-based participatory models and their coalitions are not a substitute for, nor can they provide, the population policies, legislations and regulations that enable their effective working and likelihood of more favourable outcomes. In Australia, the Commonwealth and State governments have distinct institutional obligations, instruments and means of addressing human development—they need to be asked to do different things that reflect their level of action and priorities.

Now more than ever, when governments ask “what should we do”, there are intervention options from which to choose and that offer increasing capability for customization to local or place-based circumstances (Cleary et al., 2023). These interventions are being undertaken with the express goal of enhancing the development of young children by changing their circumstances, expectations about them, and their opportunities (Taylor et al., 2020). In moving these interventions to scale their onward sustainability, in place, and more universally, we must reform the prevalent implementation environment from one of short-term feasibility trials (often with short-term special-purpose funding or leveraged funding) to one of recurrent public investment in human development (Marmot, 2010; Rose, 2001).

## Early Relational Health in Perspective

A singular focus on early relational health can pose a significant challenge for researchers: What constitutes an outcome when so much of life is ahead (Zubrick et al., 2009)?

The reviews in this series make it clear that the early foundational establishment of trust, safety and security between an infant and their closest carers matters significantly. It is a beginning of a cascade of developmental effects. These effects can certainly be seen as far as at least early maturity.

From these earliest attachment experiences emerge the later skill sets needed to establish trust, regulate emotions, engage in exploratory behaviour, communicate effectively, possess some degree of introspection and develop—to some degree—self-efficacy in meeting life’s challenges. These skills are used by individuals across the life course to influence their social and physical environment, to expand their choices, and for their own development and for the development of others.

Our contributing authors raise a challenge to think more broadly; to bring more holistic frameworks to the table that capture the breadth of the social ecology around the developing child; that make better use of universal public health service systems to promote the relational health of micro- to macrosystems; and that more fully appreciate the intergenerational legacy that shapes the full social ecology of the developing human.

We have immense challenges to face collectively, globally, now and into the future. The research presented here offers evidence to invest in these opportunities to promote, protect, and develop not just early relational health, but many of the onward skills that will be needed to meet these challenges.

## Data Availability

No datasets were generated or analysed during the current study.
